# The Role of Local Seasonal Foods in Enhancing Sustainable Food Consumption: A Systematic Literature Review

**DOI:** 10.3390/foods10092206

**Published:** 2021-09-17

**Authors:** Alexandre Maia Vargas, Ana Pinto de Moura, Rosires Deliza, Luís Miguel Cunha

**Affiliations:** 1 GreenUPorto—Sustainable Agrifood Production Research Centre/Inov4Agro, Departamento de Geociências, Ambiente e Ordenamento do Território (DGAOT), Faculty of Sciences, University of Porto, 4485-646 Vila do Conde, Portugal; up202003694@edu.fcna.up.pt; 2 Department of Gastronomy, IESB—Instituto de Educação Superior de Brasília, SGAS Quadra 613/614, Brasília 70200-730, DF, Brazil; 3 GreenUPorto—Sustainable Agrifood Production Research Centre/INOV4Agro, Departamento de Ciência e Tecnologia (DceT), Universidade Aberta, Rua do Amial, 752, 4200-055 Porto, Portugal; apmoura@uab.pt; 4 EMBRAPA Agroindústria de Alimentos, Av. das Américas, 29501, Rio de Janeiro 23020-470, RJ, Brazil; rosires.deliza@embrapa.br

**Keywords:** seasonal, local food, food chain, consumer behavior, sustainability

## Abstract

This article aims to review the current literature pertaining to the effects of eating local seasonal food on sustainable consumption. To this end, we examined definitions of seasonal and local food, the methodological approaches adopted to study the impact of seasonal consumption on sustainability, and sustainability dimensions investigated in journal articles. Highlighting what seasonal and local means, it is crucial to evaluate the effect of the consumption of these foods on sustainability. A systematic review of the literature was conducted using Scopus and Clarivate’s Web of Science database in line with the recommendations from the Preferred Reporting Items for Systematic Reviews and Meta Analyses (PRISMA) guidelines. Our findings suggest that the concept of local seasonality provides relevant information to the study of sustainable consumption. However, for better use of this concept, it is crucial to define what is local. At this point, regulation of labels based on geographic proximity or political boundaries proves pertinent.

## 1. Introduction

In the 21st century, food sustainability is at the forefront of research in agriculture and public health nutrition. This topic is a central point for discussing new policies involving food security, the environment, and food production, considering the paradigm of sustainable development [1,2].

An example of political efforts for sustainable development, a result of multilateralism and international policy shaping, is The United Nations’ (UN) 2030 Sustainable Development Agenda. Covering 17 Sustainable Development Goals (SDGs), it is a normative agenda on sustainable development, comprising of 169 targets that offer a quantitative agenda towards achieving the goals [3]. Food sustainability is the central theme of some SDGs, such as poverty eradication, ending hunger, and defending the environment.

Another policy that encompasses sustainable development is the European Green Deal, which aims to improve European citizens’ quality of life, defend nature, and transform the current economic model, for all citizens [4]. Food sustainability policies are represented in the Green Deal by the “Farm to Fork Strategy”. This approach establishes regulatory and non-regulatory proposals to make food systems fair, healthy, and environmentally friendly [5].

The focus on “food system” is elucidated by the fact that, as modern food systems production is ever more global and industrialized, it substantially influences climate-changing GHG emissions, from farming through processing, distribution, and likewise food consumption [6]. In the food sustainability challenge, the end point of the supply chain—the consumer—has become the focus of concern. In this context, “sustainability of food consumption” receives particular attention, as consumers choose the products and services they consume, and their lifestyles affect how they enforce healthy and sustainable practices [7,8].

The relations between societal, environmental, and economic development are crucial to the integral concept of sustainability. These so-called three dimensions of sustainability need to be addressed in assessing a policy or project’s sustainability. Considering the multidimensions of seasonality, Seyfang [9] elaborated a theoretical framework, based on five dimensions, to analyze sustainable consumption: localization, community building, reducing the ecological footprint, collective action, and building new provisioning infrastructures. Some of the sustainable food practices presented by previous research that considered sustainability dimensions include selecting local, organic, and seasonal food [10,11,12,13,14].

Unlike organic food, which already has a more concrete and regulated concept in many countries, the interpretation of local and seasonal food can vary depending on the context that is being used, and who is using it. When exploring the other narratives that have been produced about local and seasonal foods, there are varied understandings. For example, many consumers associate seasonal with locally produced food, but by other definitions, local is not a necessary criterion for seasonal food [15].

The definition of “local food” and its impact on sustainable consumption has long been discussed in the alternative food networks literature. The concept of local food depends on and is contextualized through the places and people wherein food is produced and consumed. There is also enormous complexity involved in understanding the sustainability of local food networks [16].

Concerning seasonal food, some articles considered the implications of seasonality on the different sustainability elements [17,18,19,20]. Those articles concluded that consuming seasonal foods as the only sustainable action has little impact on sustainable consumption.

To date, many studies relating to sustainable food consumption have focused on specific and singular sustainability-related foods, such as limiting themselves to only local or only seasonal foods. However, those approaches lack a holistic view of the subject, only researching the indirect effects of seasonal foods consumption. They do not contemplate other environmental and social considerations that need to be attempted [21].

A growing body of articles and reviews examine the environmental and socio-economic impacts of local food consumption. However, this literature has not yet yielded consistent results on how seasonality affects sustainable food consumption practices, and neither do those articles study the effects of the relationships between local food and seasonality in the sustainability of food consumption.

The concept of local and seasonal are often thought as synonyms between researchers and consumers. The understanding of the meanings of seasonal and local is essential to evaluate the impact of food consumption on sustainability. It is urgent to clarify that there is a difference in the environmental, social, and economic impact of local products when considering only the geographical concept. Farming systems are complex and multifunctional; therefore, agricultural techniques cannot be limited to a succession of single practices (even if virtuous) along the food chain. It is critical to understand the importance of an integrated vision of the processes, going beyond single cultures and field boundaries and involving the entire chain from production to consumption [22].

In this perspective, it appears necessary to carry out a critical work of systematization and organization of the existing literature, which is helpful to highlight the definition of seasonal and local food, the stakeholders’ perspective of the effects of eating local seasonal food in all dimensions of sustainability, and prominent trends that emerged in the analysis of sustainable consumer consumption.

This article aims to review the current research literature on the effects of eating local seasonal food on consumption sustainability. First, the methods of the systematic review are described. The second section brings an overview of existing studies, and reviews different concepts of seasonal and local food. The third section discusses the impact of consuming local and seasonal food on the three dimensions of sustainability. The last section suggests using the local seasonal food concept for developing new policies and strategies involving sustainable consumption, and brings recommendations for future works.

## 2. Materials and Methods

A systematic review of the literature was conducted in June 2021 using Scopus and Clarivate’s Web of Science database in line with the recommendations from the Preferred Reporting Items for Systematic Reviews and Meta Analyses (PRISMA) guidelines [23].

A search for “seasonal food” or “local food” produced 12,319 articles in June 2021. After the first attempt, the search fields were narrowed searching the two main keywords separately with: “sustainability”; “short supply chain”; “consumer”; “circular economy”. To cover both “seasonal” and “seasonality” when adding the other keywords, the term “seasonal*” was embraced. The symbol “*” broadens a search for finding words that start with the same letters. To optimize the systematic review, searches for seasonal food and local food were categorized into search 1 and search 2, respectively. The search strategy is detailed in Table 1.

The search scope was then limited to “journal articles” with the language in “English” while excluding conference papers, short surveys, notes and errata, reducing the number of articles to 9170. Original peer-reviewed articles were considered if they included aspects of the seasonal and local agri-food supply chain: definitions, member relationship, composition and governance, quality, and factors affecting sustainable development.

**Figure 1:**
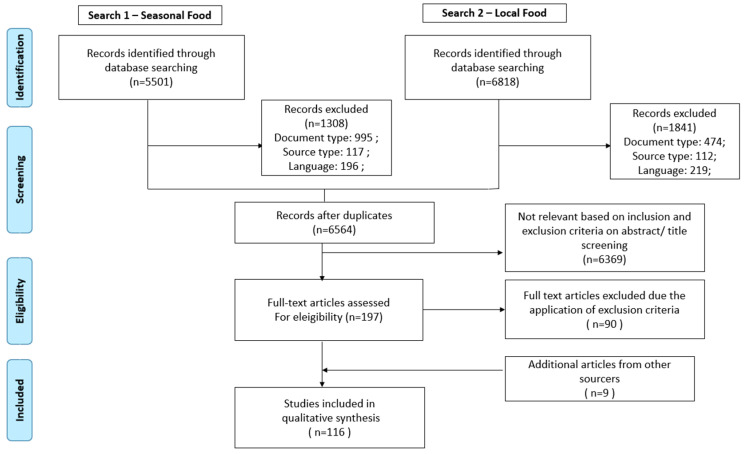
PRISMA flow diagram of the study selection. Adapted from Page et al. [23].

Literature reviews and articles using mathematical modeling or geospatial methods for local production capacity calculation and other articles that did not directly cover the local or seasonal agri-food chain were excluded. In this research, no timeframe inclusion or exclusion criteria were established. Figure 1 shows the flow diagram of the search and selection process.

Seven main factors were used to classify the different articles: (i) Authors and year of publication, (ii) Methodology, (iii) Address or Reference Seasonality, (iv) Aim, (v) Scope, (vi) Participants and sample, and (vii) Sustainability dimensions addressed (social, economic, and environmental).

The sustainability dimension analysis was based on the three dimensions of sustainability: environmental, social, and economic, usually symbolized by three interlocking circles [2]. The popular three-circles diagram appears to have been first presented by Barbier, in 1987, although the three-pillar framework was a gradual construction. Elkington’s “triple bottom line” was very influential in cementing the three-pillar position in the mainstream [24]. Although a contemporary sustainability literature may focus on the SDGs, the three pillars themselves were clearly embedded in their creation [7].

## 3. Results

### 3.1. Overview of Identified Studies

A total of 9170 articles were retrieved from database searching. After removing 3242 duplicate articles, titles and abstracts of articles were screened based on inclusion and exclusion criteria. Then, 197 articles were selected for full-text reading, and 90 were excluded using the exclusion criteria. Nine articles identified through screening references of review papers concerning local food were also included. Finally, 116 relevant studies were selected for the final analysis. The list of articles analyzed in the systematic review regarding local and seasonality concepts, ordered by publication year is available in the Appendix A.

**Figure 2:**
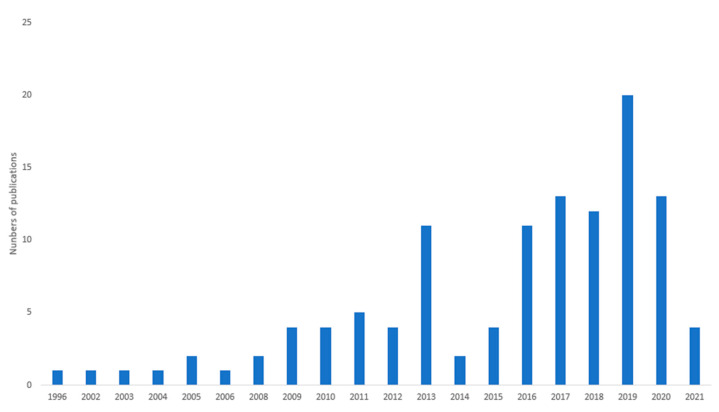
Distribution by publication year of the 116 publications included in the analysis.

Figure 2 presents an overview of the evolution of publications by year. In our sample, the first papers that openly studied seasonality were published in 1996. Since then, there has been a slow but constant increase in the number of publications addressing seasonality, and after the release of the UN SDGs in 2016, the number of articles increased further, peaking in 2019.

Concerning the sustainability dimensions, 35 articles (30.2%) focused on the economic dimension of sustainability, where most of them analyzed consumer preferences and willingness to pay for local food. The environmental sustainability was addressed in nine papers (7.7%), studying multiple attributes linked to production and distribution, such as the carbon footprint, food miles, and organic farming. Three papers (2.6%) addressed the economic and environmental dimensions of sustainability.

**Figure 3:**
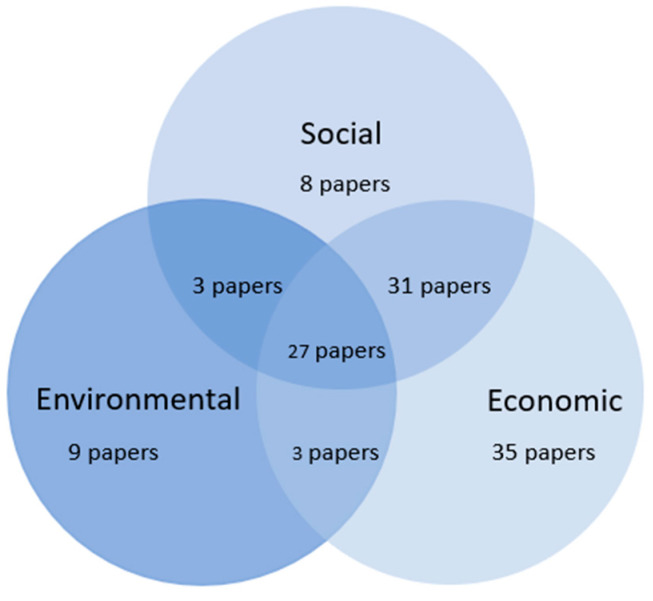
Sustainability dimensions addressed in papers of our sample.

The social dimension was the focus of eight articles (6.9%), considering attributes related to animal welfare, social responsibility, and the relationship between the local product networks’ actors. Additionally, 31 manuscripts (26.7%) addressed social sustainability and economic dimensions, while three (2.6%) reported on social sustainability and environmental dimensions. Finally, 27 out of 116 papers (23.3%) considered all social sustainability, economic, and environmental dimensions to some degree (see Figure 3).

### 3.2. Seasonal Food

In the systematic review, 34 of the 116 articles (29.3%) addressed or referenced seasonality, and only six (5.2%) directly dealt with seasonality as a central focus. Several consulted websites and papers did not give a clear description of seasonal and local concepts. As a result, there is no legal or universally recognized meaning of seasonal and local food.

One interpretation of seasonal food used in many articles is closely linked to the consumption of food produced locally, including the presumed environmental gain of less transportation [21,25,26,27,28,29]. Another understanding is the growing of vegetables and fruits in their “natural growing season” without using greenhouses [30].

A study ordered by the United Kingdom’s Department for Environment, Food and Rural Affairs (DEFRA) [18] suggested two definitions of seasonal food: Global Seasonality and Local Seasonality. In the Global Seasonality context, food is produced during the natural growing/production period for the country or region where it is produced, but it is not necessarily consumed where it is produced. In the Local Seasonality definition, food is produced outdoors without high-energy use climate modification or storage and being consumed in geographic proximity to the production.

The first DEFRA definition for seasonal food is a production-oriented approach or “global” definition, and the second is a consumer-oriented approach or “local” definition [18]. The most important element that embraces these definitions is the fact that, for both settings, food is produced outdoors in its natural season without additional energy, thus not creating additional greenhouse gas emissions [20].

Considering the literature review, three different concepts of seasonal food were found, as shown in Table 2. The first concept, called “in season”, is linked only to the availability of food. Articles that used this concept did not specify the type of production and where the food was consumed.

For the second concept, “produced in season”, seasonality is linked to availability and the food production management system. It takes seasonal food that is produced during the natural growing period without additional energy, as the DEFRA’S concept of global seasonality. Nevertheless, this approach did not determine whether this food should be consumed where it is produced.

Finally, “local seasonal” directly links the food production management system to the place of production and consumption. As a result, local seasonal foods are produced and consumed within geographical proximity, and they are growing or produced outdoors in their natural season without high-energy use climate modification or storage.

### 3.3. Local Food

The term “local food”, “local food system” or “short food supply chain” embraces various dimensions. Current definitions of local food are still too vague and contested as they have different interpretations in places with different agro-food contexts [54]. Additionally, it is important to highlight a difference between local food—one produced and consumed locally—and locality food (produced locally and consumed globally) [55].

The meaning of local food is usually related to geographic boundaries, but definitions of “local” vary regarding whether it is presented from a producer, a supplier, or a consumer point of view [56,57]. As shown in Table 3, three different concepts of local food were found: geographic, holistic, and regional.

The geographic definitions for local food were recognized in 61 out of the 116 papers (52.6%). Those definitions are based on geographic proximity or political boundaries [58,69]. These units of analysis (geographical and political) are mostly driven by population statistics and agricultural production data collected by regions, states, or countries. However, these dimensions may not include ideal units to explore more significant local food self-sufficiency opportunities, as they are rarely reflective of or coherent with bioregions [136].

There is no consensus, or a standard maximum distance between production and consumption, on when products are to be considered local food. In the U.S., for example, “local” food is not officially defined; the 2008 Farm Act determines local food as: “less than 400 miles (approximately 644 km) from its source, or within the state in which it is made” [137]. Similarly, in Europe there is no uniform definition of local food. European Agricultural Fund for Rural Development, in Regulation (EU) No 807/2014, merely gives that the definition of local markets suitable for funding by the EU shall be defined in Member States’ rural development programs [138]. In France, in 2009, the Ministry of Agriculture created the “short circuit”, an initiative that encourages the consumption of direct sales from production within less than 150 km, or through indirect sales with only one intermediary between the farmers and the consumer [139].

In this context, Kneafseg et al. [140] defined “Local Food Systems” as those where the production, processing, trade, and food consumption phases occur in a defined reduced geographical area (depending on the sources and reflections, of about 20 to 100 km radius). Meyerding et al. [15], in a consumer choice experiment developed in Germany, suggested that retailers should adopt a portfolio of local labeling schemes to meet different needs and demands of German consumers, namely: “local”, “within state”, and “within 30 km”. Granvik et al. [79] concluded in their Swedish case study that a common definition of local food is not needed as long as the individual actors are transparent with their definition.

Morris and Buller [132] noted that beyond geographic definition, “local” also represents concepts such as “specialty” and “locality” foods, creating a differentiation of the food or destination. In a consumer study developed by Wilkins et al. [25], the most frequent significance for the term “local” food was “foods grown locally”, although other dimensions were reported: “distance”, “physical accessibility”, and “specialty” or “uniqueness”.

In fact, consumers buy local food products as they are supposed to have higher quality (undergo less processing and be fresher) and to be more nutritious and healthier; to have a better flavor (emphasizing their authenticity); to support the rural areas by offering an ever-growing multiplier effect within the local economy; and to have an environmentally responsible production process [33,54]. Schmitt et al. [54] named seven principles of localness: geographical distance, supply chain size, proportion of direct sales, number of intermediaries, product identity concerning territory, local know-how, and governance.

The holistic concept of local food was identified in 60 (51.7%) of the reviewed articles. In this concept, local food is produced in geographic proximity with trust and connectedness between and within producer groups and consumers. It is represented mainly by short food supply chains and cooperative networks of consumers and producers that commonly pursue the goal to maintain traditional farming practices through new models and social improvement.

The regional concept focuses on the identity of local food. In this concept, local food is produced in geographic proximity and contains characteristics such as “specialty” and “identity” that differentiate similar foods produced in other places. Making a distinction between this concept of local food and regional or traditional food is exceedingly difficult as they are often used interchangeably. Such a situation may lead to mistakes in analyzing reasons for customers’ and producers’ behaviors [141].

For the articles selected in this review that had seasonality as a central focus, none of them utilized the holistic concept of local food. Considering the articles that addressed or referenced seasonality and used the “local season” concept, 11 out of 16 (68.7%) used the geographic definition for local food.

## 4. Discussion on Sustainability of Local Seasonal Food

The interrelationships between the environmental, societal, and economic development are central to the concept of sustainability. It is crucial to recognize the connections and interactions among these three “pillars” of sustainability to pursue sustainable development in industrialized and developing nations [11]. Sheth et al. [142] affirm that current sustainability strategies have three primary deficiencies: they fail in recognizing the looming threats from rising global over-consumption, they do not directly focus on the customer, and do not use a holistic approach.

Eating seasonal food is being promoted as one aspect of a sustainable diet, frequently interpreted as local food, but the social, environmental, and economic benefits and limitations need to be compared with supplying year-round fresh produce [20]. The seasonal marketing of food is becoming gradually more popular, while at the same time the food systems in the developed world have increasingly eliminated seasonality [143].

The expansion of global supply chains has been reinforced by the prospects of the year-round availability of seasonal fresh food products and the comparative advantage of some geographies for specific food production [140]. In contrast, Marchetti et al. [22] affirmed that it is essential to valorize complex bioecological, functional, economic, and social interactions that embody farming systems. The support of diversified farming systems is one of the possible solutions to increase productivity and at the same time foster the social-ecological transition.

Urban Agriculture (UA) has been discussed as an alternative to a greater supply of local products in urban regions. UA is increasing all over the world, especially in projects inside and on urban buildings: e.g., rooftop farms, rooftop greenhouses, and plant factories. These UA projects, characterized by the non-use of land or acreage for farming activities, are represented by some authors by the term “Zero-Acreage Farming”’ (ZFarming) [144,145].

The most noteworthy distinction between UA and rural agriculture is the integration of urban agriculture into the urban economic system and urban ecosystems [146]. UA has flourished in some cases, such as community gardening, as a collective movement that seeks to address various economic, social, and environmental challenges [118]. On the other hand, Specht et al. [145] affirmed that it is vital to recognize that different types of ZFarming are not in and of themselves sustainable, and the practices of those projects can be as unsustainable as conventional agribusiness.

Graamans et al. [147] analyzed the performance of plant factories compared with cultivation in traditional greenhouses by analyzing the use of resources in the production of lettuce, concluding that plant factories may offer many advantages, such as a quality of production, but have a high energetic demand due to the need for artificial lighting. For UA projects to be sustainable, they need to focus on energy-efficient production, building new market structures, local resources, and involving the social dimension [145].

Ending hunger and all forms of malnutrition are central themes of the SDGs agenda [3]. Eating wild food is often touted as the mechanism to stave off hunger when food shortages are present [64]. Wild edible plants are seasonal foods that are still consumed by a large section of the global population, and they ensure affordable food and nutritional security [148]. Although those plants are commonly consumed frequently, for most families, this frequency increases during periods of food scarcity [149].

The availability of wild edible plants is very dependent on seasonality, besides the weather and access to the surrounding where they grow naturally. This variable availability driven by seasonality and climate extremes means that these foods may not be available in sufficient quantities when required [149]. In countries where wild foods continue to be part of the routine cuisine, these foods can be replaced for less expensive foods with less cultural meaning and produced far from its consumption [38].

### 4.1. Economic and Social Dimensions

Concerning the economic dimension, most of the articles included in this systematic review analyzed consumers’ preferences and willingness to pay for local food. Hempel and Hamm [150] showed that many studies revealed a high consumer preference and willingness to pay for local food. Werner et al. [87] concluded that consumers participating in the survey favored supporting the local economy, but were only willing to pay price premiums for a few specific local produce options. Moreno and Malone [135] found a positive relationship between collective food identity and consumer preferences for localness; however, the consumer was only willing to pay price premiums for products that are largely locally produced. Although the products studied by Moreno and Malone in the discrete choice experiment were fruits, it is essential to highlight the differences between local food and local products.

Some products are “produced” locally, but not necessarily from local ingredients. Jams and bread are prime examples of this. For instance, Milestad et al. [35] concluded that the local organic cereal and bread network actors in Lower Austria were reluctantly dependent on the “global” food system. One of the most used criteria for putting manufactured products into analyzed categories is the place of production [106]. Making a distinction between this concept of local food and local product may be crucial to analyze consumers’ preferences and willingness to pay.

In an experiment using locality labels in watermelons, Bernard et al. [91] showed that the addition of the region label increased the consumer’s willingness to pay and expectations that the watermelon would taste better and would be safer. The local indication proposed further tangible representations of the food product in terms of how it is produced, positively influencing the consumer’s evaluations [51]. Although those labels enhance local products’ trust, they only referred to the geographical concept of locality. This approach may restrict and hinder the complexity of the different actors along the local food chain. Birtalan et al. [53] affirmed that the term “local food” also brings a particular emotional perspective to consumers’ minds and the idea of building social interactions.

Telligman et al. [81] investigated beef consumers in grocery stores across Alabama (U.S.) and found that consumers most commonly understand local beef as a product originating within a specific geographic boundary, particularly the State. It is noteworthy that almost a quarter of the participants had multidimensional meanings for local beef. Besides geographic definitions, consumers characterized local beef by the production practices and relationships with producers, e.g., “Especially from here, you’re familiar with the farm.”; “… it is someone you know; you know what they feed, you know how they take care of the cows”. A trust-building marketing strategy that combines the existing positive associations with local beef production with a connection to the territory is promising to rise the consumption of these products [57].

The consumer decision-making process may be a result of conscious choices among an array of alternatives, and many socio-cognitive models interpret consumer choice as a method of problem-solving determined by rational information processing (e.g., Value–Belief–Norm theory and Attitude–Behavior–Context theory) [65,151]. Consumption is a social, cultural, and economic process of choosing goods. A deeper understanding of consumption requires this multidimensional perspective, which envisages consumption as a holistic process [152,153]. It is important to highlight that willingness to pay (WTP) is relevant as a proxy for individual preferences, but it is criticized as it contains limited information [154].

Darolt et al. [49] describe a long process of empowering consumers and making them conscious of factors such as the seasonality of ecological production. In addition, transmuting the local food system towards sustainability involves precise framing. Presently, distinct groups of actors use similar structures but with different meanings [96]. On the other hand, traditional social networks have limited scope to suggest and affect distant actors [42].

Consequently, a transition to sustainability is characterized as a systemic change of the interrelated immaterial and material elements, rather than purely a change of consumer behaviors [108]. Furthermore, the increasing distance between production and consumption is a rising concern for consumers [41,155]; therefore, the need to set up metrics, such as indicators, to assess food systems’ sustainability has been stressed [156]. Using “food-miles” as an indicator of environmental sustainability, a measure of how far food travels after production towards consumption has been a consistent debate on food sustainability articles [54,60].

Currently, there has been growing discussion about the role of food and the human-centered interaction and behavior around food. Models of short food supply and Alternative Foods Networks (AFN) are shifting toward an arena where the food topic is linked to other urban policy fields, sectors, and functions, such as quality of life, health, social inclusion, urban renewal, and education [119]. In this context, CSAs give a good platform for consumers and farmers to narrow their interactions. They are also developing a fundamental approach to increase sustainable agriculture and food consumption [29]. The expansion of CSAs depends on citizens’ awareness of environmental protection, involving their comprehension and implementation capacity of circular economy, environmental policy, and green production [124].

### 4.2. Environment Dimension

The food system’s environmental impact is multidimensional, with implications for water use, climate change (i.e., GHGE), biodiversity, land use, pollution, and soil degradation. However, there are very limited studies that have explored all these issues simultaneously, concerning seasonality, as most have focused on GHGE and climate change [20]. Schmitt et al. [75] selected a set of attributes and indicators of performance to compare the multi-dimensional performance of a local food chain with a global one. The authors concluded that the global chain might have the advantage of emitting fewer GHG emissions per kilo of milk produced, and might be more efficient in terms of production costs as farmers in the global chain showed higher annual income, but the local initiative of the study is still in its beginning.

Brooks et al. [17] reported that even for a single food, adjustments in the seasonal production pattern can affect the environmental impacts of food production by influencing the scale, timing, and type of fertilizer or pesticide applied. Studying the use of agricultural chemicals, Schoolman [80] showed the results of a two-way, fixed effects regression model indicating that, in the USA, growth in local food systems, whether measured as an increase in the number of farms selling direct market products or as an increase in the total value of direct market sales, was strongly associated with declines in spending on agricultural chemicals in 1997. Across the country, however, the magnitude of this relationship steadily decreased over the following 15 years. One possible explanation proposed by the author is that as the social movement for local food gathered steam after 1997, it increasingly attracted producers, consumers, and marketing outlets that did not necessarily prioritize quasi-organic or low-input farming practices.

The concept of food miles has provided an important political and ideological role in emphasizing the significance of carbon footprints in the food system [61]. However, concentrating on how “distance” or “food miles” can make consumption more sustainable has been criticized for shifting the debate away from sustainable agricultural production and focusing purely on food distribution [61,156,157].

The term “local trap” has gained strength by showing the lack of compelling evidence that local means sustainable or fair. Born and Purcell [104] affirmed that the local trap assumes that a local-scale food system will be inherently more socially fair than a national-scale or global-scale food system. Dupuis and Goodman [157] alerted to the problem of associating the local food term with quality or sustainability, as a local product can be grown with exploitation of families, workers, or the environment.

Life cycle assessment (LCA) has been widely used to evaluate the environmental impact and identify more sustainable agricultural production options [158]. The environmental impact categories assessed by LCA were carbon footprint, water footprint, fossil fuel and mineral resource depletion, acidification, eutrophication, and ecotoxicity from pesticide use. However, few life cycle analyses of food products or systems explicitly explored seasonality. When LCA referred to “seasonality”, the term was associated with a crop’s “natural growing season” [19].

Röös and Karlsson [30] concluded that the results of the carbon footprint of carrot and tomato consumption were strongly affected according to either a strict definition of seasonality, which excluded both long-distance transport and production in heated greenhouses, or a definition which only allowed Swedish produce. The high-energy use needed for lighting and heating to grow products out of season can have higher GHGE than the emissions associated with transportation [19].

Based on the results of case studies, Brooks et al. [17] affirmed that applying DEFRA’s local seasonality definition, which incorporates a local link between production and consumption, e.g., produced and consumed in the same climatic zone without high energy use for growing or storage, would be more likely to deliver environmental benefits than applying the “global” definition. The authors concluded that a critical limitation of all definitions of seasonal food as a guide to environmental impact is the impossibility of unraveling local influences on the impact of seasonal shifts in the local production.

Thus, it is important to build the social and ecological sustainability transition as a multidimensional concept, which goes far beyond the “local market” or “seasonal food” dimension only. A multi-dimensionality framework avoids falling into “the local trap” and may be used for exploring new practices of food consumption, where reducing the ecological footprint and strengthening local economies are just some of the elements in a more complex process of changing the market structure and infrastructure [108].

This is especially important as this multidimensional understanding of sustainability must compete with a growing discourse of economic regionalism that focuses on local economic production without necessarily integrating the ecological and social dimensions [115]. For example, a tomato from a local high input large-scale production can be endorsed with a “regional” label. However, such local label is not related to sustainable consumption and/or production methods or even produced respecting local seasonality.

## 5. Conclusions

The analysis of the 116 selected studies suggests that local seasonality is an appropriate concept for the investigation of sustainable consumption. However, it is crucial to define what is local for adequate use of this concept. The definition of “local food” and its impact on sustainable consumption has long been explored in the literature. It is not possible to assume that a local food will have a smaller impact than global food simply as it was produced close to consumption. On the other hand, it is not possible to ensure that a global food is more sustainable than a local one considering only a smaller carbon footprint.

As an alternative to binary thinking, where “local” and “global”, “local seasonality” and “in season” are distinct from each other, we recommend a systemic view of the food system. This may permit a more complex analysis where food actors may be locally orientated and globally connected. Adopting a holistic concept of “local food” or embracing a “local seasonal food” concept can force methodological approaches that address all the pillars of sustainability, allowing more concrete results towards a sustainable consumption. Future works that study the relations between the local seasonal food chain actors using a multi-dimensionality framework can bring relevant results.

We recognize limitations in our research as not all possible sources of information were brought to the review for the established inclusion and exclusion criteria, the database, and selection of keywords. Furthermore, the authors’ potential subjectivity during the thematic analysis could be also seen as a limitation.

It is important to draw attention to the limited number of articles addressing all dimensions of sustainability and involving more than two stakeholders. We encourage efforts to study the impact of consumption of local seasonal food considering the point of view of all main stakeholders. Furthermore, we highlight the importance of investigating the sustainability of local and seasonal food in developing countries, which seems largely ignored in the journals we considered.

Despite the need for a holistic view in new policies and strategies involving sustainable consumption, label regulation based on geographic proximity or political boundaries must be considered public policy to increase the consumption of local seasonal products. Creating labels for local food can help create a standardized distance between production and consumption that will help create a solid concept of what it means to provide local food and local seasonal food. Although, it proves necessary to respect social, economic, and environmental characteristics when delimiting the maximum distance between production and consumption to pursue sustainable consumption.

## Figures and Tables

**Table 1:** Search strategy with presentation of the keywords used to evaluate information about seasonal and local food. The symbol “*” broadens a search for finding words that start with the same letters, such as “seasonality”.

Database		Search Strategy
Scopus and Web of Science	Search 1 #1	Seasonal food
	Search 1 #2	Seasonal* AND Sustainability
	Search 1 #3	Seasonal* AND Short Supply Chain
	Search 1 #4	Seasonal* AND Consumer
	Search 1 #5	Seasonal* AND Circular Economy
	Search 2 #1	Local food
	Search 2 #2	Local food AND Sustainability
	Search 2 #3	Local food AND Short Supply Chain
	Search 2 #4	Local food AND Consumer
	Search 2 #5	Local food AND Circular Economy

**Table 2:** Identified concepts and relative definitions used for seasonal food.

Concepts	Definition	References
In season	Linked only to natural food availability e.g., “uncertainties of seasonality and weather in production planning is the primary requirement of economically viable farming” [31]	[14,21,25,30,31,32,33,34,35,36,37,38,39,40,41,42,43]
Produced in Season	Linked simultaneously to availability and type of production. Food that is produced in their natural growing season, without high energy use for climate modification. e.g., “The range of fresh products is made available either through imports from countries where the growing season is longer or occurs at a different time of the year or through energy-demanding technologies that extend the normal growing season, predominantly heated greenhouses” [29]	[15,17,19,30,44]
Local Seasonal	Linked simultaneously to availability, location, and type of production. The food is produced and consumed within geographical proximity. It is produced outdoors in its natural growing season, without high energy use for climate modification or storage. e.g., “CSA programs encourage local production and consumption by allowing consumers to subscribe to a membership and, in return, receive food periodically from a group of local farmers during the harvest season” [28]	[17,19,21,26,27,28,29,30,45,46,47,48,49,50,51,52,53]

**Table 3:** Identified concepts and relative definitions use for local food.

Concepts	Definition	References
Geographic	Food is produced in a geographic proximity or in a specific political boundary, e.g., Tomato from within a 50 km radius or a German Tomato	[15,17,19,21,25,26,27,28,30,32,33,34,38,41,46,47,51,54,56,57,58,59,60,61,62,63,64,65,66,67,68,69,70,71,72,73,74,75,76,77,78,79,80,81,82,83,84,85,86,87,88,89,90,91,92,93,94,95,96,97,98]
Holistic	Food produced in geographic proximity with trust and connectedness between and within producer groups and consumers. It is mainly represented by short food supply chains and cooperative networks of consumers and producers that commonly pursue to maintain traditional farming practices through new models and social improvement; e.g., Community-Supported Agriculture (CSA)	[14,22,29,35,36,37,39,40,42,43,45,48,49,50,51,52,53,54,56,79,90,97,99,100,101,102,103,104,105,106,107,108,109,110,111,112,113,114,115,116,117,118,119,120,121,122,123,124,125,126,127,128,129]
Regional	Food that represents concepts such as “specialty” and “identity”, containing a differentiation of the food, e.g., Parma Ham	[7,75,76,122,126,130,131,132,133,134,135]

## Appendix A

**Table A1:** List of articles analyzed in the systematic review regarding local and seasonality concepts, ordered by publication year.

Author and Year of Publication	Aim	Sustainable Dimensions	Methodology	Address or Reference Seasonality	Scope	Sample	Identified concept of Seasonality *	Identified concept of Local Food **	Reference
Wilkins, 1996	To explore the relationship between a preference for local foods and other dietary patterns.	Social, Economic, and Environment	Mailed survey	Yes	Seasonal and Local Food	N = 309 Puget Consumers’ Cooperative (PCC) members (n = 193) and a random selection of Washington State (US) residents (n = 116)	IS	G	[32]
Wilkins et al., 2002	To explore how consumers conceptualize “local” and “seasonal” as applied to foods.	Social, Economic and Environment	Face-to-face survey open-ended questions about the meanings of local and seasonal foods	Yes	Seasonal and Local Food	N = 120 Shoppers in a grocery store and a food cooperative	IS	G, R	[25]
Morris and Buller, 2003	To investigate the range and scope of local food production in the county of Gloucestershire and consider the potential of local food production and marketing for adding value for the various actors in the chain.	Economic	Case Study and face-to-face interviews	No	Local Food	N = 23 Farmers (n = 15); retailers (n = 8)	N.A.	R	[132]
Zepeda and Leviten-Reid, 2004	To investigate consumers’ interests, attitudes, and motivations for buying local food.	Economic and Environment	Focus Group	No	Local Food	N = 41 Alternative food shoppers (n = 22); Conventional consumers (n = 21)	N.A.	G	[58]
Roininen et al., 2005	To establish the personal values, meanings, and specific benefits consumers relate to local food products.	Social and Economic	Word association and laddering interviews	No	Local Food	N = 55 Consumers	N.A.	H	[103]
Selfa and Qazi, 2005	To exam how consumers and producers conceptualize local.	Social and Economic	Case Study and online survey	Yes	Local Food	Food Chain Key Informants	IS	G	[33]
Born and Purcell, 2006	To theorize geographical scale that entirely precludes the local trap.	Social, Economic, and Environment	Theoretical approach to scale local trap	No	Local Food	Theoretical approach	N.A.	H	[104]
Sirieix et al., 2008	To identify whether food miles matter to French consumers.	Social, Economic, and Environment	Focus groups and face-to-face interviews	No	Food Miles	N = 26 Random consumers (n = 16) for a focus group; consumers of locally grown organic food consumers for survey (n = 10)	N.A.	G	[59]
Weber and Mattews, 2008	To compare the life-cycle greenhouse gas (GHG) emissions associated with food production against long-distance distribution.	Environment	Life Cycle Assessment	No	Food Miles	N.A.	N.A.	G	[60]
Coley et al., 2009	To discuss the conception of food miles, followed by an empirical application of food miles to two contrasting food distribution systems based on carbon emissions accounting within these systems.	Environment	Data analysis of fuel and energy use from one UK’s supplier of organic produce	No	Food Miles	N.A.	N.A.	G	[61]
Cross et al., 2009	To compare the self-reported health of farm workers who were producing the same product in four different countries with relevant population norms.	Social	Health-related quality of life approach using survey instrument adapted from SF-36, EuroQuol EQ-5D, and Visual Analogue Scale (VAS)	No	Worker Health in local and global food systems	N = 2545 food systems workers	N.A.	G	[62]
Nousiainen et al., 2009	To examine factors that contribute to the social sustainability of AFS, by focusing on local and organic food system initiatives in Juva, Finland.	Social	Case study and face-to-face interviews	No	Alternative Food Networks	N = 20 AFN stakeholders	N.A.	G, H	[63]
Zepeda and Deal, 2009	To increase understanding of why consumers buy organic and/or local foods.	Economic	Face-to-face interviews	No	Local Food	N = 25 Consumers	N.A.	G	[64]
Arnoult et al., 2010	To estimate willingness to pay for foods of a designated origin, together with certification for organic and free of genetically modified (GM) ingredients.	Economic	Focus groups and face-to-face interviews; choice experiment	Yes	Seasonal Food	N = 222 Consumers	IS	G	[34]
Conner et al., 2010	To identify opportunities and obstacles which inform marketing strategies for local food and farmers markets and reflect the demographic diversity of the state.	Economic	Telephone survey	No	Local Food	N = 953 Consumers	N.A.	G	[65]
Louden and MacRae, 2010	To examine whether current federal labeling rules might impede the marketing of local and sustainable claims.	Economic	Case study; data analysis on current local and sustainable food labeling regulation and application	No	Value-added labels	N.A.	N.A.	G	[66]
Milestad et al., 2010	To explore the social relations between food actors and how “local” and “organic” are expressed by detailing how actors describe qualities of their intra-network relationships, how they understand “local”, and how they are connected within the food system.	Social and Economic	Face-to-face interviews and workshops	Yes	Local Food	N = 15 Food Chain Key Informants	IS	H	[35]
Bean and Sharp, 2011	To examine how two possible consumptive pathways, the purchase of organic foods and/or the purchase of local foods, can affect food sustainability.	Economic	Mailed survey	No	Local and Organic Food	N = 2398 Consumers	N.A.	G	[67]
Brooks et al., 2011	To summarizes research by Defra, which studied consumers’ understanding of, and attitudes to, seasonal foods, and the environmental implications of applying certain “seasonal” definitions to guide food sourcing.	Social, Economic, and Environment	Focus groups, online quantitative survey, and Life Cycle Assessment	Yes	Seasonal Food	N= 1200 Grocery buyers	PS, LS	G	[17]
Levidow and Psarikidou, 2011	To explore agro-food relocalization initiative	Economic and Environment	Case study and face-to-face interviews	No	Local Food	N = 12 Local food chain stakeholders	N.A.	H	[105]
Pearson et al., 2011	To analyze of consumers of a local food retail outlet in the UK that is based on weekly community markets.	Social, Economic, and Environment	Case study; face-to-face survey	Yes	Local Food	N = 183 Consumers	LS	H	[45]
Wirth et al., 2011	To determine the relative strength of two credence attributes, organic and locally produced, within the context of fresh apple.	Economic	Focus groups and online survey; conjoint analysis	No	Local and Organic Food	N = 1218 Consumers	N.A.	G	[68]
Hayden and Buck, 2012	To identify whether and how CSA membership affects environmental ethics.	Economic	Online survey and face-to-face interviews	No	CSA	N = 48 CSA members	N.A.	H	[106]
Hu et al., 2012	To estimate consumer willingness to pay for varieties of a processed food product that are differentiated with respect to their local production labelling and a series of other value-added claims.	Social and Economic	Mailed survey; conjoint analysis	No	Value-added labels	N= 1884 Consumers	N.A.	G	[69]
Rainbolt et al., 2012	To identify factors that might influence consumer valuation of organic, fair trade, and local labeled food.	Economic	Online survey	No	Local Food	N = 1269 Consumers	N.A.	G	[70]
Uribe et al., 2012	To examine whether ecological attitudes of CSA members could predict food and sustainability related behaviors.	Social and Environment	Online survey	Yes	CSA	N = 115 CSA members	IS	H	[36]
Amate and González De Molina, 2013	To evaluate the energy cost of the Spanish agri-food (AFS) system in the year 2000 with a view to ascertaining the relative importance of each link in the agri-food chain.	Environment	Life Cycle Assessment	Yes	Agroecological Food System	N = 1 Country (Spain)	LS	G	[46]
Aubry and Kebir, 2013	To investigate the role of SSFCs in a potential revival of the food supply function of agriculture located close to cities.	Social and Economic	Face-to-face interview	No	Short Food Supply Chains	N = 68 Short supply food chains stakeholders; consumers (n = 90); farmers (n = 60); Decision-makers (n = 8)	N.A.	H	[107]
Fonte, 2013	To examine the discourses and practices of GAS (*Gruppi di Acquisto Solidali*) operating in Rome (Italy).	Social, Economic and Environment	Face-to-face interviews	No	Solidarity Purchasing Groups	N = 28 Solidarity Purchasing Groups Representatives	N.A.	H	[108]
Long and Murray, 2013	To explores convergence and divergence of ethical consumption values through a study of organic, fair trade, and local food consumers in Colorado	Economic	Mailed survey and focus groups	No	Ethical consumption	N = 469 Consumers	N.A.	H	[109]
Pratt, 2013	To analyses a small island ecotourism project in Fiji in the context of food miles and sustainability.	Social and Economic	Online survey	Yes	Food Miles	N = 205 Consumers	LS	G	[47]
Röös and Karlsson, 2013	To investigate how the carbon footprint of yearly per capita consumption of tomatoes and carrots in Sweden was affected by seasonal consumption according to interpretations of seasonality found in communications from Swedish NGOs and authorities.	Environment	Data analysis of carbon footprint of yearly per capita consumption of tomatoes and carrots in Sweden was affected by seasonal consumption according to interpretations of seasonality	Yes	Seasonal Food and Carbon Footprint	N.A.	IS, PS, LS	G	[30]
Schnell, 2013	To explore the ongoing debates over food miles and local food.	Social, Economic and Environment	Face-to-face interviews	Yes	Food Miles and CSAs	N = 30 CSA members	IS	H	[37]
Sneyd, 2013	To analyze wild food consumption in urban areas of Cameroon.	Social and Economic	Face-to-face interviews	Yes	Wild Food	N = 371 household and market’s consumers	IS	G	[38]
Wang et al., 2013	To identify the patterns of main meal preparation among Australian adult household meal preparers and the relationships between these patterns and likely socio-demographic and psychological predictors.	Economic	Online survey	Yes	Patterns of main meal preparation	N = 222 Consumers	IS	-	[31]
Echeverría et al., 2013	To elicit the WTP of Chilean consumers towards the carbon footprint of food products, controlling for several consumer’s attributes.	Economic and Environment	Contingent valuation; double bounded dichotomous choice survey	No	Carbon footprint on foods	N = 774 Supermarket consumers	N.A.	G	[71]
Foster et al., 2013	To explore the environmental implications of upstream changes that arise as supply of particular foodstuffs progresses through the year.	Environment	Life Cycle Assessment	Yes	Seasonal Food	Data analysis	PS, LS	G	[19]
Knutson et al., 2014	To analyze the public role of trade of U.S. fresh fruit and vegetable demand.	Economic	Data analysis of prices and quantities for fruits and fresh vegetables in USA from 1970 to 2011; vector autoregression	Yes	Fresh Fruit and Vegetable Demand	N = 1 Country (United States of America)	PS		[44]
Lillywhite and Simonsen, 2014	To evaluate consumers’ locally produced ingredient preferences relative to the price of the dining experience and restaurant type.	Social and Economic	Online survey; conjoint analysis	No	Local Food	N = 320 Consumers	N.A.	G	[72]
Cleveland et al., 2015	To analyze the origin and effects of the focus on spatial scale and build on this analysis by operationalizing the concept of “local” food.	Social, Economic, and Environment	Case study; data analysis of public comments associated with reducing food miles as an action for and indicator of alternative food systems	No	Local Food	N = 496 comments	N.A.	G, H	[73]
Dedeurwaerdere et al., 2015	To analyze the governance features of local food buying groups by comparing 104 groups in five cities in Belgium.	Economic	Face-to-face interviews	No	Local Food	N = 104 Collective Food Buying Groups Members	N.A.	H	[115]
Favilli et al., 2015	To analyze the innovation potential of a local food network.	Social	Case Study; action research and participatory approach; workshops and face-to-face interviews	No	Organic Food	N = 20 Organic Food Systems stakeholders	N.A.	H	[116]
Memery et al., 2015	To investigate how attributes associated with local food (intrinsic product quality; local support) motivate purchase behavior.	Economic	Online survey	No	Local Food	N = 1223 Consumers	N.A.	H	[117]
Aprile et al., 2016	To examine consumers’ perception, attitude, and motivations for buying local foods and identify profiles of local foods’ consumers.	Economic	Face-to-face survey	No	Local Food	N = 200 Consumers	N.A.	G, H	[74]
Balázs et al., 2016	To examine how farmer led CSA movement in Hungary creates an alternative in the dominant food regime.	Social and Economic	Face-to-face semi-structured interviews; a consumer-member survey and secondary data sources were utilized; the data analysis used thematic coding.	Yes	CSA	N = 91 Producers (N = 5); Policy makers and experts (n = 3); CSA Members (n = 83)	IS	H	[39]
De Boer et al., 2016	To explore how the transition to a low-carbon society to mitigate climate change can be better supported by a diet change.	Social, Economic, and Environment	Online survey; profile analysis was used to assess how the participants evaluated the mitigation options.	Yes	Carbon Footprint	N = 1083 Consumers from the Netherlands (n = 527) and the United States (n = 556)	LS	G	[26]
Chiffoleau et al., 2016	To explore the conditions under which local food chains in urban food systems can bring about an evolution in the practices and knowledge of “ordinary” actors with no or limited skills in agriculture and/or awareness of sustainability.	Social, Economic, and Environment	Case Study; face-to-face interviews	Yes	Short Food Supply Chain	N = 60 Retailers (n = 30); Consumers (n = 30)	LS	H	[48]
Darolt et al., 2016	To show that alternative food networks produce social innovation, diversity, and new values that can contribute to reconnect producers and consumers, aggregate value to local markets through short distribution channels.	Social, Economic, and Environment	Face-to-face interviews	Yes	Alternative Food Networks	N = 20 Food Chain Key Informants	LS	H	[49]
Hvitsand, 2016	To identify why Norwegian producers and consumers engage in CSA and how CSA can be seen as a transformational act toward food system changes.	Social, Economic, and Environment	Face-to-face interviews with farmers; online survey with CSA members	Yes	CSA	N = 456 Farmers (n = 7); Consumers (n = 449)	LS	H	[50]
Merle et al., 2016	To examine the impact of a local origin label on perceptions and purchase intent regarding food products.	Social, Economic, and Environment	Online survey	Yes	Indication of Local Geographic Origin	N = 509 Consumers	LS	G, H, R	[51]
Mundler and Laughrea, 2016	To evaluate the contributions of SFSCs to territorial development in three contrasting Quebec territories.	Social, Economic, and Environment	Case Study; online survey	No	Short Food Supply Chains	N = 97 Short supply food chains stakeholders	N.A.	H	[110]
O’Kane, 2016	To examine the individual, social, physical, and macro-level environments that can positively or negatively influence peoples’ engagement with food citizenship.	Social and Economic	Focus groups; narrative inquiry	Yes	Food Citizenship	N = 52 Community gardeners (n = 6); regular farmers’ market shoppers (n = 10); CSA members (n = 4); fresh food market (n = 8); and supermarket shoppers (n = 24)	IS	H	[14]
Schmitt et al., 2016	To compare the multi-dimensional performance of a local with a global food chain.	Social, Economic, and Environment	Selection of a set of attributes and indicators of performance to compare the multidimensional performance of a local with a global food chain; face-to-face interviews	No	Local and Global milk chain	N = 10 Local milk stakeholders (n = 6); Global milk stakeholders (n = 4)	N.A.	G, R	[75]
Touzard et al., 2016	To objectivize which aspects of wine are local, and which are global, using a multidimensional analytical approach.	Social, Economic, and Environment	Case study; analytical and participatory approach; focus groups and face-to-face interviews	No	Local and Global wine	N = 24 Local wine chain stakeholders	N.A.	G, R	[76]
Bakos et al., 2017	To present an overview of the development and current state of CSAs systems on the international and Hungarian level.	Social and Economic	Face-to-face survey	Yes	CSA	N = 817	IS	H	[40]
Bellante, 2017	To explore innovative strategies and limitations of AFNs.	Social and Economic	Case study and face-to-face interviews	No	Alternative Food Networks	N = 51 Local Food Systems stakeholders	N.A.	H	[111]
Berg and Preston, 2017	To address the question of which observable factors about consumers are relatively important in influencing expenditures, shopping frequencies, and willingness to pay (WTP) premiums for local food.	Economic	Face-to-face interviews	No	Local Food	N = 237 Farm Market Consumers	N.A.	G	[77]
Bianchi, 2017	To examine the drivers of local food purchase intentions for Chilean consumers	Economic	Online survey	No	Local Food	N = 1000 Consumers	N.A.	G, H	[78]
Granvik et al., 2017	To contribute to knowledge on definitions, interpretations, and practices of local food by presenting views and opinions among different actors in the food chain in a Swedish context.	Social and Economic	Online survey	No	Local Food	N= 158 Local Food Systems stakeholders from: Sweden (n = 97); Austria (n = 2); Great Britain (n = 23); Denmark (n = 1); Finland (n = 1); Italy (n = 3); Norway (n = 1); Spain (n = 1); The Netherlands (n = 6); and USA (n = 22)	N.A.	G, H, R	[79]
Lurie and Brekken, 2017	To analyze the contribution of small-scale agriculture in rural Oregon to the framework.	Economic	Online survey	No	New natural resource economy	N = 642 Farmers (n = 153); Consumers (n = 489)	N.A.	H	[112]
Lutz et al., 2017	To illustrate various forms of cooperation in relation to small-scale farming and the establishment of local food supply.	Social and Economic	Case study; Social Multi-Criteria Evaluation and workshops	No	Local Food	N = 6 Farmers	N.A.	H	[113]
Russel and Zepeda, 2017	To examine attitude and behavior change associated with CSA membership.	Social and Economic	Focus groups	No	CSA	N = 23 CSA members	N.A.	H	[114]
Schmutz et al., 2017	To build a more detailed understanding of different types of urban SFSC and their relative performance compared to each other.	Social and Economic	Case study; sustainability impact assessment; workshop and face-to-face interviews	No	Short Food Supply Chains	N = 86 Short supply food chains stakeholders	N.A.	H	[119]
Schoolman, 2017	To explore whether growth in local food systems is associated with decreased on-farm use of agricultural chemicals.	Environment	Longitudinal data analysis from the US Census of Agriculture to explore whether growth in local food systems is associated with decreased on-farm use of agricultural chemicals.	No	Local food	N.A.	N.A.	G	[80]
Telligman et al., 2017	To investigate U.S. consumers’ perceptions of local beef, including the definitions and types of quality perceptions held for local beef products.	Social, Economic, and Environment	Face-to-face interviews	No	Local beef	N = 174 Beef consumers	N.A.	G, H	[81]
Tichenor et al., 2017	To quantify the environmental burdens of grass-fed beef with management-intensive grazing and confinement dairy beef production systems in the northeastern U.S.	Environment	Life cycle assessment	No	Beef production systems	N.A.	N.A.	G	[82]
White and Bunn, 2017	To identify a series of emergent policy pathways for UA practice and demonstrate that local government can assume a diverse leadership role as a promoter, enabler, and manager of UA.	Social and Environment	Case study; participant observation; focus groups and face-to-face interviews.	No	Urban agriculture	N = 30 Urban Agriculture actors	N.A.	H	[118]
De Chabert-Rios et al., 2018	To understand the reasons why some restaurateurs are entering the farming business, and to learn about the financial, operational, and customer-related benefits and challenges encountered by restaurateurs operating their own farms.	Economic	Case study and face-to-face interviews	Yes	Local Food	N = 3 Restaurants	LS	G	[27]
Crawford et al., 2018	To understand the socio-demographic characteristics and motivations, concerns, and attitudes of shoppers attending farmers’ markets in Sydney.	Social	Face-to-face survey	Yes	Local Food	N = 633 Farmers’ Markets Consumers	LS	G	[28]
Furman and Papavasiliou, 2018	To examine how a food hub with close ties to the local food movement in Atlanta, Georgia contends with this issue as it articulates with larger markets.	Social and Economic	Case study; participatory action research; face-to-face interviews	No	Food Hubs	N = 34 Food hub managers (n = 5); farmers (n = 21); chefs (n = 8)	N.A.	H	[120]
Hashem et al., 2018	To explain the growing interest of English consumers in local organic food sold through box schemes.	Social, Economic, and Environment	Face-to-face interviews; online survey	No	Local and Organic Box Schemes	N = 438 Box scheme consumers	N.A.	H	[122]
Kulick, 2018	To explore how one statewide food network in the United States seeks to involve youth contending with the juvenile justice system in a job readiness program.	Social	Case study; participatory action research; face-to-face interview	No	Alternative Food Networks	N = 24 Stakeholders in the local food (n = 7) and juvenile justice system (n = 17)	N.A.	H	[99]
McKay et al., 2018	To examine restaurant WTP for local products.	Economic	Telephone survey; contingent valuation	No	Local Food	N = 152 Restaurants	N.A.	G	[83]
Pícha et al., 2018	To test the parameters that influenced preferences among food products branded as national, regional, or local products.	Social and Economic	Face-to-face survey	No	Local Food	N = 988 Consumers	N.A.	G, H, R	[90]
Scalvedi and Saba, 2018	To identify sustainability aspects that overlap with local and organic consumer profiles in order to provide evidence that can be used to promote both kinds of foods in a sustainable food consumption (SFC) integrated framework.	Social and Economic	Face-to-face survey	Yes	Local and Organic Food	N = 3004 Consumers	N.A.	G, H	[21]
Schmitt et al., 2018	To address the lack of metrics for quantifying the degree of localness of a food value chain (FVC) using a multi-criteria evaluation.	Social, Economic, and Environment	Case study and face-to-face interviews	No	Local Food	N = 97 Local (n = 11) and global (n = 86) cheese chain stakeholders	N.A.	G, H, R	[54]
Skog et al., 2018	To investigate how adaptive governance of LFS can provide ideas and act as a catalyst for creating resilience in other social-ecological systems.	Social and Economic	Case study and face-to-face interviews	No	Local Food	N = 20 Local Food Systems stakeholders	N.A.	G, H	[95]
Tookes et al., 2018	To examine the interplay between demand for local and ethically sourced foods and the implications for seafood sustainability in the U.S. south.	Social, Economic, and Environment	Face-to-face survey	Yes	Local Seafood	N = 500 Farmers market shoppers	LS	H	[52]
Vitali et al., 2018	To assess greenhouse gas (GHG) emissions associated with a local organic beef supply chain using a cradle-to-grave approach.	Environment	Life Cycle Assessment	No	Local Organic Beef	N.A.	N.A.	G	[84]
Baldy, 2019	To identify how local actors are framing the food system and what this means for increasing sustainability.	Social, Economic, and Environment	Case study; participatory observation; workshops and face-to-face interviews	No	Local Food	N = 26 Local Food Systems stakeholders	N.A.	G	[96]
Beingessner and Flecher, 2019	To examine local food systems from the producer perspective in a rural context of high industrialization and geographical dispersion.	Social and Economic	Case study; face-to-face interviews and focus groups	No	Local Food	N = 60 Local Food Systems stakeholders	N.A.	H	[100]
Bernard et al., 2019	To examine the impacts of minimal-information labels using field experiments with watermelons.	Economic	Face-to-face interviews	No	Value-added labels	N = 328 Consumers	N.A.	G	[91]
Bryla, 2019	To assess the level and predictors of regional ethnocentrism on the market of regional food products in the context of sustainable consumption.	Economic	Computer-assisted web interview	No	Regional Ethnocentrism	N = 1000 Consumers	N.A.	R	[133]
Chen et al., 2019	To analyze how the motivation, barriers, and methods of advertisement influence the participation dynamics of CSA by segmenting consumers based on their past, current, and future CSA participation.	Social and Economic	Online survey	Yes	CSA	N = 795 Consumers	N.A.	H	[29]
Corsi and Mazzocchi, 2019	To assesses the agricultural and territorial drivers that influence the development of AFNs.	Social	Data analysis of the factors influencing the participation of consumers and farmers in AFNs using an Ordinary Least Squares (OLS) regression	No	Alternative Food Networks	N.A	N.A	H	[128]
Denver et al., 2019	To investigate the preferences and trade-offs of distinct consumer segments relative to organic production and several dimensions of local food.	Economic	Online survey; choice experiment	No	Local and Organic Food	N = 505 Consumers	N.A.	G	[85]
Fan et al., 2019	To assess the effect of locally grown information on consumer WTP and quality perceptions of three broccoli varieties.	Economic	Sensory evaluation of broccoli using affective test	No	Local Food	N = 240 Consumers	N.A.	G	[86]
Meyerdin et al., 2019	To explore whether consumers prefer specific local food labeling strategies to others, and where there is a difference between fresh and processed tomatoes.	Economic	Face-to-face to survey; choice-based conjoint analysis	Yes	Local Food	N = 640 German Consumers	PS	G	[15]
Nakandala and Lau, 2019	To investigate the characteristics of demand and supply in relation to the real-world supply chain strategies of local urban fresh food supply chains.	Social and Economic	Case study; face-to-face interviews	Yes	Urban fresh food supply chains	N = 12 Urban local fresh food retailers	IS	G	[41]
Nicolosi et al., 2019	To analyze the preferences of Swedish consumers for local/artisanal cheeses and the purchase motivations that guide their choices.	Social	Face-to-face interviews; social network analysis	No	Local Food	N = 200 Consumers	N.A.	H, R	[122]
Olson et al., 2019	To assesses the impact of the local food economy in Hardwick using environmental, economic, and social outcomes.	Social, Economic, and Environment	Face-to-face interviews	No	Local Food	N = 21 Local Food Systems stakeholders	N.A.	R	[131]
Osei-Owusu et al., 2019	To assesses the global cropland footprint of Danish food and feed supply.	Environment	Data analysis assessing the global cropland footprint of Danish food and feed supply from 2000 to 2013 using a consumption-based physical accounting approach	No	Footprint	N.A.	N.A.	G	[92]
Paul, 2019	To investigate if a new model of farming—CSA—is delivering sustainable livelihoods to farmers.	Social and Economic	Face-to-face interviews	No	CSA	N = 14 CSA farmers	N.A.	H	[122]
Profeta and Hamm, 2019	To analyze if a local feed origin labelling is a promising strategy to accompany the efforts being made in the production of local feedstuffs.	Social and Economic	Computer self-assisted personal interviews; discrete-choice experiment	No	Local Food	N = 1602 Consumers	N.A.	H	[101]
Santo and Moragues-Faus, 2019	To examine the trans-local dimension of food policy networks and its potential to facilitate transformative food system reform.	Social and Economic	Case study; participant observation; face-to-face interviews	No	Local Food	N = 22 Trans-local food policy networks projects members	N.A.	G	[98]
Tang et al., 2019	To identify the experiences and current problems and demonstrating recent research and development status of CSA in China.	Social, Economic, and Environment	Data analysis of CSA status in China	No	CSA	Data analysis	N.A.	H	[124]
Tatebayashi et al., 2019	To identify the structure of food-sharing networks and non- market food species.	Social	Face-to-face interviews and online survey	Yes	Food Sharing Network	N = 281 Farmers (n = 15); consumers (251)	IS	H	[43]
Werner et al., 2019	To identify key factors of understanding local food systems from a regional perspective.	Social and Economic	Online and mailed survey; discrete choice analysis; data analysis of farmland acreage	No	Local Food	N = 756 Restaurants (n = 109); consumers (n = 647)	N.A.	G	[87]
Witzling and Shaw, 2019	To understand local food consumers and take steps to begin to identify how targeted messages could engage different groups.	Economic	Mailed survey	No	Local Food	N = 577 Consumers	N.A.	H	[102]
Bareja-Wawryszuk, 2020	To analyze of the forms of organization and spatial concentration of local food systems in Poland.	Economic	Data analysis based on the register of local entities to identify and characterize the forms of organization of local food systems in Poland	No	Local Food	N = 1067 Consumers	N.A.	G	[88]
Barska and Wojciechowska-Solis, 2020	To identify the behavior of Polish consumers shopping online for local food products and to identify barriers to purchase.	Economic	Online survey	No	Local Food	N = 1067 Consumers	N.A.	G, H	[97]
Birtalan et al., 2020	To explore food-related well-being among CSA members.	Social and Economic	Face-to-face interviews; thematic analysis	Yes	CSA	N = 35 CSA members	LS	H	[53]
Bisht, 2020	To explore the effects of the implementation of sustainability practices to traditional farming and food systems.	Social and Economic	Focus-group	No	Local and Organic Food	N = 1000 Farmers	N.A.	H	[125]
Fogarassy et al., 2020	To explore the circular characteristics of consumers’ attitude towards food purchasing in Hungary.	Economic	Face-to-face interviews	No	Circular Economy and Organic Food	N = 828 Consumers	N.A.	H	[129]
Horská et al., 2020	To identify the factors that influence the sales of local products with a focus on value-added dairy products.	Social and Economic	Case study; face-to-face interviews	No	Short Food Supply Chains	N = 30 Family Farms	N.A.	H	[127]
Kopczyńska, 2020	To compare the collectives based on novel alternative food networks and traditional networks.	Social, Economic, and Environment	Face-to-face interviews	Yes	Local Food	N = 38 Consumers	IS	H	[42]
Ku and Kan, 2020	To examines the potential contribution of social work intervention in responding to China’s agrarian challenges.	Social and Economic	Case study; participatory action research	No	Local Food	N = 347 Households	N.A.	H, R	[126]
Marchetti et al., 2020	To present and denounce environmentally and socially unsustainable agricultural practice, which cause negative effects on environment, health, and social and intergenerational equity.	Social, Economic, and Environment	Grounded theory approach; desk analysis to detail some directions and perspectives about food production systems and sustainability	No	Food Production System	N.A.	N.A.	H	[22]
Oravecz et al., 2020	To identify the main characteristics of Hungarian consumer preferences when buying honey.	Economic	Face-to-face interviews	No	Local Food	N = 1584 Consumers	N.A.	G	[89]
Printezis and Grebitus et al., 2020	To investigate the preferences and willingness to pay of college student millennials for unprocessed (fresh) or processed (typically come in a container) food products sold at urban farms.	Economic	Online survey; choice experiment	No	Urban Agriculture	N = 443 College Students	N.A.	G	[93]
Sanjuán-López and Resano-Ezcaray, 2020	To analyze the willingness to pay for the local, organic, and PDO (Protected Designation of Origin), their differences across experimental conditions, and by identifying the effects of personal characteristics.	Economic	Face-to-face interviews; choice experiment with the Random Utility Model	No	Local Food	N = 208 Consumers	N.A.	R	[134]
Tremblay et al., 2020	To identify and describe the dietary importance of different wild animal species across the range of communities included and assess the extent to which dietary diversity correlates with geography, culture, and ecology.	Social and Environment	Case study and face-to-face interviews	No	Local Food	N = 21 Indigenous communities	N.A.	R	[130]
González-azcárate et al., 2021	To achieve a better understanding of SFSCs in terms of potential market niches, key food choice attributes, and perceived barriers and drivers.	Social, Economic, and Environment	Face-to-face and random telephone survey	No	Short Food Supply Chains	N = 1969 Active SFSC consumers (N = 394); potential SFSC consumers (N = 422) and the general public in Spain (N = 1153).	N.A.	G	[94]
Kim and Huang, 2021	To develop a novel framework of local food consumption which explores the dynamic relationships among local food ideology.	Economic	Online survey	No	Local Food	N = 297 Consumers	N.A.	G, H, R	[56]
Kuhl et al., 2021	To analyze how an ideal local beef production should be constituted.	Economic	Online survey	No	Local Beef	N = 432 Consumers	N.A.	G	[57]
Moreno and Malone, 2021	To explore the interaction between local food identity and agricultural production.	Economic	Online survey; discrete choice experiment	No	Local Food	N = 484 Consumers	N.A.	R	[135]

* Concepts of seasonal food: IS—In season; PS—Produced in season; LS—Local seasonal. ** Concepts of local food: G—Geographic; H—Holistic; R—Regional; N.A—Not applicable.
